# Clinical characteristics and treatment management of combined large cell neuroendocrine carcinoma, a subtype of large cell neuroendocrine carcinoma

**DOI:** 10.3389/fonc.2024.1449490

**Published:** 2024-10-22

**Authors:** Kai Kang, Binfeng Li, Sheng Wang, Jianjian Wang, Xinjun Liang

**Affiliations:** ^1^ Thoracic Surgery, Hubei Cancer Hospital, Tongji Medical College, Huazhong University of Science and Technology, Wuhan, Hubei, China; ^2^ Department of Abdominal Oncology, Hubei Cancer Hospital, Tongji Medical College, Huazhong University of Science and Technology, Wuhan, Hubei, China

**Keywords:** combined large cell neuroendocrine carcinoma, LCNEC, molecular typing, RB1 and TP53 co-mutation, adjuvant chemotherapy

## Abstract

Combined large cell neuroendocrine carcinoma (CLCNEC) is a rare neuroendocrine carcinoma, accounting for approximately 10% of large cell neuroendocrine carcinoma (LCNEC). Mainly composed of coexisting adenocarcinoma components, with strong invasiveness and poor prognosis. The treatment regimen for CLCNEC mainly refers to complete surgical resection as the first choice in the early stage, while patients with stage II or higher require adjuvant treatment. At present, research on CLCNEC is mostly small sample and retrospective, and there is no consensus on whether molecular typing and treatment should be carried out. There is considerable controversy over whether it should be managed as small-cell lung cancer (SCLC) or non-small-cell lung cancer (NSCLC). Therefore, in order to solve the problem of confusion in the selection of treatment regimens for CLCNEC, while also considering the therapeutic effects, this article summarizes and analyzes previous studies, fully seeks evidence, and boldly proposes new therapeutic insights: the etoposide-platinum (EP) regimen serves as the basis for adjuvant therapy; In addition, SCLC/NSCLC-CLCNEC can be distinguished based on presence of RB1 and TP53 co-mutation, and targeted therapy or NSCLC type chemotherapy including platinum + gemcitabine or taxanes (NSCLC-GEM/TAX) can be used in combination or sequentially for NSCLC-CLCNEC.

## 1 Rare and uncontrollable CLCNEC

Lung large cell neuroendocrine carcinoma is a relatively rare cancer with high metastasis rate and short survival period, accounting for about 3% of all lung cancers (1–3). International Agency for Research on Cancer (IARC) classification of Thoracic Tumors (5th Edition) published in 2021 classified pulmonary combined large cell neuroendocrine carcinoma (CLCNEC) as a subtype of LCNEC associated with LCNEC components and epithelial components such as adenocarcinoma or squamous carcinoma. In practice, >10% of LCNEC patients are diagnosed with combined LCNEC (CLCNEC) (4, 5). According to reports, CLCNEC is more aggressive and has a poorer prognosis than LCNEC (6, 7). At present, there are relatively few case reports and small-scale retrospective studies on CLCNEC. The main research still analyzes all components as a whole, without distinguishing between pure or combination components (8, 9). According to previous studies, the incidence of CLCNEC is related to males, middle-aged and elderly individuals, and severe smoking (8). The main symptoms include cough, expectoration, chest pain, hemoptysis, and dyspnea (10). The most common mixed component in CLCNEC is adenocarcinoma (AD), accounting for about 70%, followed by squamous cell carcinoma (SCC), accounting for 20% (6, 11). A study suggests that adenocarcinomas-LCNEC is more common in young non-smokers, with lesions typically peripherally located and accompanied by driver gene mutations. Squamous-LCNEC is more common in male patients aged 65 and above, and the lesion is close to the hilum of the lungs. In addition, there is no significant difference between disease-free survival (DFS) and overall survival (OS) between the two CLCNEC subtypes (12).

## 2 Accurate and precise diagnosis is the foundation of CLCNEC treatment

Travis et al. first uncovered LCNEC as a distinct subtype with neuroendocrine (NE) differentiation but different morphological features compared to small cell lung cancer (SCLC) (13). The pathological manifestations of LCNEC part are complex and varied, and accurate diagnosis requires observation of cell and tissue morphology through light microscopy, combined with immunohistochemical (IHC) features and neuroendocrine particles under electron microscopy. The pathological diagnostic criterion for LCNEC classified by WHO in 2021 are as follows: 1) neuroendocrine morphology with organoid nesting, palisading, rosette-like structures and granular chromatin; 2) High mitotic rate >10 mitoses per 2 mm2 (average 60–80 mitoses per 2 mm2); 3) abundant necrotic tissue; 4) NSCLC cytological features; 5) Positive immunohistochemistry for at least one neuroendocrine marker such as chromograninA(CgA), synaptophysin (Syn), neural cell adhesion molecule 1 (NCAM1/CD56) or NSE, or electron microscopy (14–17). However, the diagnosis of LCNEC is difficult on small biopsy or cytological samples, and immunohistochemistry requires the use of at least two sets of neuroendocrine markers, while CLCNEC is more prone to misdiagnosis due to the presence of other mixed components (18–20).

Researches (11, 12) have found that tumor cells are arranged in a palisade like or chrysanthemum like cluster in the part of CLCNEC, with prominent nuclei and multinucleate division, which is generally consistent with LCNEC. CLCNEC also has epithelial-derived IHC markers corresponding to the expression of focal adenocarcinoma and squamous cell carcinoma components. For example, adenocarcinoma expresses NapsinA, while squamous cell carcinoma expresses CK5/6, p40, and p63. Therefore, apart from meeting the pathologic diagnostic criteria for LCNEC, the diagnosis of CLCNEC also requires the components of adenocarcinoma, squamous cell carcinoma, giant cell carcinoma, and/or spindle cell carcinoma (16, 21). CLCNEC was classified as a subtype of LCNEC in the 2021 WHO Classification of Thoracic Tumours (15), belonging to a different family from NSCLC with NE features such as carcinoid tumor (16). Pathologically these tumors are primarily distinguished based on the mitotic counts, presence or absence of necrosis, and cytologic features (16). Typical carcinoids (TC) show < 2 mitoses per 2 mm2 and absence of necrosis, while atypical carcinoids (AC) show 2 - 10 mitoses and/or punctate foci of necrosis (15). LCNEC has a mitotic count exceeding 10 per 2 mm2, and the degree of necrosis and cellular atypia is much greater than that of that of carcinoid tumors (22). And, the necrosis of LCNEC is common and often represents extensive necrosis (23). Ki-67 staining shows a low proliferation rate in TC, usually less than 5% while in AC it is higher, usually between 5% and 20% (16). Patients with LCNEC have a higher Ki-67 index than those with TC and AC (24), with over 30% usually indicating LCNEC (25–28). At present, routine pathological examination of surgical specimens is an unquestionable diagnostic method for LCNEC, and the proposal of molecular typing can aid in diagnosis and also help discover any potential combination components (15, 18).

## 3 Molecular characteristics are the “wind vane” of CLCNEC treatment

Genomically, LCNEC is known to share frequent alterations in RB1 and TP53 with SCLC, although different frequencies are reported (4, 29–34). This also indicates that LCNEC has the same TP53 and RB1 mutations as SCLC at the individual gene level, of course, the frequency of RB1 mutations in the LCNEC is lower than that in SCLC (4). Some studies have also mentioned that high-level LCNEC and SCLC exhibit similarity in genome maps (4), with SCLC-like LCNEC having a higher Ki-67 rate and a closer morphological feature spectrum to SCLC than nsclc-like LCNEC (29). Milione et al. reported that LCNECs with co-mutation of TP53 and RB1 (SCLC-like) were significantly enriched in cases with a Ki-67 ≧55%, while the tumors with KRAS mutations were enriched in cases with Ki-67 <55% (35). These also confirms that LCNEC has a mixed genome and transcriptome profile, and a therapeutic strategy targeting only one tumor component may be ineffective (36).

In a next-generation sequencing (NGS) analysis of LCNEC, Rekhtman and colleagues identified two major molecular subgroups and one minor subgroup. The major subgroups include co-mutation/loss of TP53 and RB1 and other SCLC-type alterations, including SCLC-like LCNEC with MYC amplification, and NSCLC-like LCNEC lacking simultaneous TP53 and RB1 alterations and almost ubiquitous NSCLC-type mutations (SKT11, KRAS, and/or KEAP1) (29). In George’s research, it was also found that LCNECs are composed of two mutually exclusive subgroups - Type I with STK11/KEAP1 alterations and Type II with RB1 alterations (31). In addition, Lazaro and colleagues (37), as well as Simbolo et al. (38), have demonstrated the importance of distinguishing RB1 mutations for the development of LCNEC. At present, the co mutation of TP53 and RB1 may be an important marker for distinguishing between SCLC like and NSCLC like LCNEC, and provide a basis for subsequent treatment.

CLCNEC is a subtype of LCNEC and therefore has its molecular characteristics. Due to the presence of NSCLC part in CLCNEC, the probability of driver gene mutations is higher compared to LCNEC (4, 39). Wang Y et al. (11) conducted NGS analysis on 70 CLCNEC patients who underwent adjuvant chemotherapy after surgery and found that there were 18 patients with EGFR mutation and 4 patients with anaplastic lymphoma kinase (ALK) mutation. Yang Z et al. (12) analyzed 60 CLCNEC resected samples and found 17 patients with EGFR mutation, 4 patients with ALK rearrangement and 2 patients with KRAS mutation. In Simbolo et al.’s (36) study, the probability of KRAS mutation reached 27.8%. In another study (4), 5 out of 10 CLCNEC patients were found to have driver gene alterations. A few studies have also discovered that some CLCNEC patients have rare driver gene mutations, with an EGFR mutation rate of 8.33% and an ALK rearrangement rate of 5.77% (5). In addition, EGFR mutation, BRAF V600E mutation and KIF5B/RET fusion mutation have also been reported in cases of CLCNEC (40–42). Patients with driver gene mutations in CLCNEC were identified through NGS analysis, while some studies did not conduct NGS analysis on the majority of patients (11, 12). In the era of precision medicine, the application of NGS analysis in CLCNEC should be expanded, which can not only distinguish between SCLC like and Nsclc like CLCNEC, but also benefit patients with driver gene mutations from targeted therapy.

## 4 Specific treatment measures for CLCNEC

In recent decades, the treatment of LCNEC has been more inclined to use non-small cell lung cancer (NSCLC) regimens including cisplatin or SCLC based regimens (3, 43), which are also recommended by the guidelines of the American Society of Clinical Oncology (44). However, the treatment effect and disease remission rate are not satisfactory. For early-stage resectable LCNEC patients, surgical treatment is the primary choice, and lobectomy or pneumonectomy with a systematic nodal dissection can prolong survival (45). Retrospective studies have shown that surgical treatment can achieve satisfactory results to other NSCLC in the early stages of the tumor, and the perioperative mortality rate is also lower (46). However, the 5-year survival rate of stage III LCNEC patients is less than 30%, and the presence of lymph node metastasis is closely related to poor 5-year survival rate (47). Therefore, radical surgery is recommended, but it seems insufficient for the treatment of resectable LCNEC (48–50). Some small sample retrospective studies have shown that perioperative chemotherapy has been shown to bring survival benefits to LCNEC patients. There is controversy over whether to undergo perioperative chemotherapy for resectable stage I LCNEC (51–54), while patients with stage II or higher LCNEC (including CLCNEC) are clearly benefiting from adjuvant chemotherapy (12, 55). For the selection of perioperative chemotherapy regimen, platinum-based adjuvant chemotherapy is superior to non platinum adjuvant chemotherapy (48, 54, 56, 57), and the specific chemotherapy regimen should also be personalized based on molecular typing results.

## 5 EP regimen chemotherapy as the basis for CLCNEC as adjuvant therapy

In 2005, Rossi et al. convincingly demonstrated for the first time that the use of SCLC-based standard regimens as adjuvant chemotherapy for LCNEC patients is effective and significantly improves survival rates (3). In subsequent studies, chemotherapy regimens similar to SCLC have shown significant efficacy in treating LCNEC patients (57–59), with a clinical response rate of up to 70% (60, 61). Zhuo M et al. (62) demonstrated that the use of the etoposide-platinum regimen has advantages in treating SCLC-like LCNEC patients compared to the pemetrexed-platinum and gemcitabine/taxane-platinum-platinum regimens.The research results of Wang et al. (63) also support the viewpoint of Zhuo et al. (62) They divided 12 patients into a consistent group (NSCLC-like LCENC (n=4) treated with NSCLC-based treatment or SCLC-like LCNEC (n=8) treated with SCLC-based treatment) and an inconsistent group based on molecular subtypes. The conclusion shows that the OS of the consistent group is significantly longer than that of the inconsistent group (median 37.7 vs. 8.3 months; p=0.046). Shen YC et al. also presented that etoposide-platinum doublets acted as an independent prognostic factor for OS (5).

Recent clinical studies have reported that the prognosis of LCNEC receiving SCLC chemotherapy regimen is more effective than NSCLC (3, 57, 58, 64), whether it is in patients with pure LCNEC or CLCNEC (12). CLCNEC should be managed in a multidisciplinary setting, confirming the adjuvant chemotherapy (especially the SCLC regimen) paramount importance to improve patients’ outcome (65). A study specifically targeting patients with surgically resectable LCNEC combined with AD and LCNEC combined with SCC included 96 patients (12). Among 78 patients who received 4 cycles of adjuvant chemotherapy, 35 received etoposide based SCLC regimen, and 43 received NSCLC regimen, including pemetrexed, gemcitabine, taxanes and vinorelbine, with or without platinum. The results showed that the DFS and OS of stage II/III patients who chose the SCLC regimen were better than those who chose the NSCLC regimen. This indicates that CLCNEC patients should still receive basic treatment as neuroendocrine carcinoma patients.This was a specialized study on resectable CLCNEC. Although few patients in their cohort received NGS, it also suggests that all CLCNEC patients should be treated as neuroendocrine cancer patients.

## 6 NSCLC like CLCNEC should be treated with both NSCLC like regimens. (EP plus NSCLC GEM/TAX)

The SCLC-like subset exhibits SCLC-like morphology characterized by RB1+TP53 coalteration and responds to SCLC chemotherapy regimens, while the nsclc like subset does not. The molecular subtype classification reflected the heterogeneity of pulmonary LCNEC, which might lead to complex chemotherapy efficacy reported by previous studies, as different subtypes might predispose the patients to show different therapeutic response (66, 67). Two single-arm phase II trials in LCNEC (n=29 and n=30) showed an objective response rate (ORR) for etoposide or irinotecan combined with cisplatin ranging from 31% to 47% (58, 64), substantially lower compared to SCLC phase III trials evaluating etoposide-cisplatinum chemotherapy (ORR ≈66%) (68). A recent study was suggested that there is an increase in overall survival (OS) in advanced LCNEC patients when NSCLC regimens are adopted, especially gemcitabine-platinum rather than pemetrexed-platinum (media 7.8 vs. 5.9 months; p=0.019) and etoposide–platinum (SCLC regimens) (media 7.8 vs. 6.7 months; p=0.035) (69). In addition, an evaluation of 26 LCNEC patients showed a significantly lower overall survival for platinum–etoposide chemotherapy compared to a combination of NSCLC regimens (70). After dividing LCNEC subgroups through molecular typing, studies have also found that wild-type RB1 patients who received NSCLC-GEM/TAX treatment had significantly longer OS than those who received SCLC-PE treatment (30). This requires us to particularly consider LCNEC molecular subtypes when selecting treatment options, distinguishing NSCLC subgroups for targeted therapy or other NSCLC-based therapies. Some cases of LCNEC carrying EGFR gene activation mutations have been reported to have good efficacy against EGFR-tyrosine kinase inhibitors (TKIs) (71–75), and ALK inhibitors have also been effective in treating LCNEC cases carrying ALK rearrangements (76). Due to its NSCLC part, driver mutations are more frequent in C-LCNEC compared to pure LCNEC (4, 39), making targeted therapy play a more important role in the treatment of CLCNEC.In Wang Y et al.’s study (11), a total of 9 CLCNEC patients who developed distant metastasis after surgery received TKI treatment. Among them, 5 patients with EGFR 19del/L858R mutation and 4 patients with ALK mutation had an ORR of 66.7%. In Yang Z et al.’s study (12), there were also 5 patients with EGFR mutations and 4 patients with ALK rearrangement who received first or second generation EGFR-TKI and crizotinib treatment, respectively. All patients benefited from targeted therapy. Other case reports have found that CLCNEC patients with ALK fusion benefit from treatment with either alectinib or crizotinib (77–80). These data indicate that combination targeted therapy is the most feasible treatment option for CLCNEC patients diagnosed with non-small cell carcinoma components.

For CLCNEC without driver oncogene aberrations, there is no corresponding “molecular targeted” drug for treatment, and the combination of NSCLC-like chemotherapy is currently the available option. Initially, it was confirmed in an *in vitro* study that VP-16 is a compound that is suitable for combination with gemcitabine if used on the right schedule, especially for lung cancer patients (81). The EP regimen of chemotherapy combined with RT followed by a three cycles docetaxel was popularized after the completion of Southwest Oncology Group (SWOG) 9504, which also resulted in the optimal survival period for stage III NSCLC (82). In recent studies (83, 84), the efficacy of combination chemotherapy for different types of tumors also supports the “hypothesis” - combining multiple, individually effective, chemotherapeutic mechanisms could overcome tumor heterogeneity, producing longer lasting remissions in more patients, and perhaps even cures (85, 86).

Recently, in Wang’s study (87), NSCLC-like (without TP53/RB1 co-alterations) LCNEC patients were also attempted to receive NSCLC plus SCLC chemotherapy regimen. Although there is a lack of prospective research, combined NSCLC-based regimens is necessary for CLCNEC patients with non small cell carcinoma components when there is no driver mutation.

## 7 Radiotherapy and immunotherapy for CLCNEC are still being explored

There are few studies on radiotherapy for LCNEC patients. Some studies have shown that radiotherapy has potential benefits in improving the survival of non metastatic LCNEC patients (88), while others have shown that radiotherapy significantly improves the overall survival of stage III patients (89). Prelaj et al. found in their study of 28 patients with stage III-IV LCNEC who received chest radiotherapy and prophylactic cranial irradiation (PCI) that both mOS and mPFS were higher in patients receiving chest radiotherapy and PCI than those who did not receive treatment (90). In Rieber et al.’s study (91), brain radiotherapy was chosen as the preferred treatment for patients with LCNEC brain metastases.

More exploration is needed to determine whether ICIs can bring benefits to LCNEC patients. In a retrospective study (92), 11 PD-L1 positive LCNEC patients received treatment with nivolumab or pembrolizumab, and 60% of patients achieved partial remission. Dudnik et al. (93) found in their study of advanced LCNEC patients that ICIs can improve both quality of life and mOS. In the study by Oda et al. (94), a patient with multiple postoperative relapses of LCNEC showed a reduction in all metastatic lesions after nivolumab treatment and maintained a partial response for 5 years after surgery. The use of immunotherapy for CLCNEC patients is currently only a few case reports. Xu et al. (95) reported a case of CLCNEC patient who underwent adjuvant chemotherapy, radiotherapy and maintenance therapy with durvalumab and achieved complete remission. Another elderly male patient with CLCNEC achieved partial response after receiving chemotherapy plus atezolizumab (96).

## 8 Conclusion

CLCNEC is a relatively rare neuroendocrine carcinoma with strong invasiveness and poor prognosis. Due to the presence of other mixed components, small biopsy or cytological diagnosis is difficult, and routine pathological examination of surgical specimens is an unquestionable diagnostic method. For early-stage resectable LCNEC patients, surgical treatment is the main choice, while patients with stage II or higher CLCNEC require adjuvant treatment. In the era of precision medicine, the application of molecular spectrum analysis in CLCNEC should be expanded, whether it is distinguishing between SCLC like and Nsclc like CLCNEC through TP53 and RB1 co mutations, or searching for oncogene aberrations to perform corresponding molecular targeted therapies. There is sufficient research evidence for LCNEC patients to receive basic treatment as neuroendocrine carcinoma patients. On this basis, we believe that CLCNEC with co mutations of RB1 and TP53 should still be treated with EP regimen chemotherapy for neuroendocrine carcinoma; However, the absence of RB1 and TP53 co mutations in CLCNEC, which is purely treated as neuroendocrine carcinoma, often does not benefit patients due to the dominance of its non small cell carcinoma components, requiring the combination of adjuvant targeted therapy or NSCLC type chemotherapy including NSCLC-GEM/TAX for personalized treatment. Currently, the available studies regarding CLCNEC treatment lacks authority, and further research is needed to design larger prospective studies to confirm the effectiveness of distinguishing the components of CLCNEC for treatment. We will be committed to such studies.

## References

[1] Fasano M Della Corte CM Papaccio F Ciardiello F Morgillo F. Pulmonary large-cell neuroendocrine carcinoma: from epidemiology to therapy. J Thorac Oncol. (2015) 10(8):1133–41. doi: 10.1097/jto.0000000000000589 PMC4503246 26039012

[2] Hendifar AE Marchevsky AM Tuli R. Neuroendocrine tumors of the lung: current challenges and advances in the diagnosis and management of well-differentiated disease. J Thorac Oncol. (2017) 12(3):425–36. doi: 10.1016/j.jtho.2016.11.2222 27890494

[3] Rossi G Cavazza A Marchioni A Longo L Migaldi M Sartori G. Role of chemotherapy and the receptor tyrosine kinases KIT, PDGFRalpha, PDGFRbeta, and Met in large-cell neuroendocrine carcinoma of the lung. J Clin Oncol. (2005) 23(34):8774–85. doi: 10.1200/JCO.2005.02.8233 16314638

[4] Miyoshi T Umemura S Matsumura Y Mimaki S Tada S Makinoshima H. Genomic profiling of large-cell neuroendocrine carcinoma of the lung. Clin Cancer Res. (2017) 23(3):757–65. doi: 10.1158/1078-0432.ccr-16-0355 27507618

[5] Shen Y Hu F Li CH Xu JL Zhong RB Zhang XY. Clinical features and outcomes analysis of surgical resected pulmonary large-cell neuroendocrine carcinoma with adjuvant chemotherapy. Front Oncol. (2020) 10:556194. doi: 10.3389/fonc.2020.556194 33335851 PMC7736707

[6] Zhang JT Li Y Yan LX Zhu ZF Dong XR Chu Q. Disparity in clinical outcomes between pure and combined pulmonary large-cell neuroendocrine carcinoma: A multi-center retrospective study. Lung Cancer. (2020) 139:118–23. doi: 10.1016/j.lungcan.2019.11.004 31775086

[7] Cakir E Demirag E Aydin M Unsal E. Clinicopathologic features and prognostic significance of lung tumours with mixed histologic patterns. Acta Chir Belg. (2009) 109:489–93. doi: 10.1080/00015458.2009.11680466 19803261

[8] Isaka M Nakagawa K Ohde Y Okumura T Watanabe R Ito I. A clinicopathological study of peripheral, small-sized high-grade neuroendocrine tumours of the lung: differences between small-cell lung carcinoma and large-cell neuroendocrine carcinoma. Eur J Cardiothorac Surg. (2012) 41(4):841–6. doi: 10.1093/ejcts/ezr132 22219429

[9] Wang J Ye L Cai H Jin ML. Comparative study of large cell neuroendocrine carcinoma and small cell lung carcinoma in high-grade neuroendocrine tumors of the lung: a large population-based study. J Cancer. (2019) 10(18):4226–36. doi: 10.7150/jca.33367 PMC6691699 31413741

[10] Sanchez de Cos Escuin J. Diagnosis and treatment of neuroendocrine lung tumors. Arch Bronconeumol. (2014) 50(9):392–6. doi: 10.1016/j.arbres.2014.02.004 24685201

[11] Wang Y Qian F Chen Y Yang ZY Hu MJ Lu J. Comparative study of pulmonary combined large-cell neuroendocrine carcinoma and combined small-cell carcinoma in surgically resected high-grade neuroendocrine tumors of the lung. Front Oncol. (2021) 11:714549. doi: 10.3389/fonc.2021.714549 34631540 PMC8493068

[12] Yang Z Wang Y Chen Y Qian FF Zhang YW Hu MJ. Combined large cell neuroendocrine carcinoma: clinical characteristics, prognosis and postoperative management. Eur J Cardiothorac Surg. (2022) 62(2):ezaco69. doi: 10.1093/ejcts/ezac069 35147672

[13] Travis WD Linnoila RI Tsokos MG Hitchcock CL Cutler GB Jr Nieman L. Neuroendocrine tumors of the lung with proposed criteria for large-cell neuroendocrine carcinoma. An ultrastructural, immunohistochemical, and flow cytometric study of 35 cases. Am J Surg Pathol. (1991) 15(6):529–53. doi: 10.1097/00000478-199106000-00003 1709558

[14] Travis WD Brambilla E Burke AP Marx A Nicholson AG. Introduction to the 2015 world health organization classification of tumors of the lung, pleura, thymus, and heart. J Thorac Oncol. (2015) 10(9):1240–2. doi: 10.1097/jto.0000000000000663 26291007

[15] Nicholson AG Tsao MS Beasley MB Borczuk AC Brambilla E Cooper WA. The 2021 WHO classification of lung tumors: impact of advances since 2015. J Thorac Oncol. (2022) 17(3):362–87. doi: 10.1016/j.jtho.2021.11.003 34808341

[16] Travis WD. Pathology and diagnosis of neuroendocrine tumors: lung neuroendocrine. Thorac Surg Clin. (2014) 24(3):257–66. doi: 10.1016/j.thorsurg.2014.04.001 25065926

[17] Rossi G Longo L Barbieri F Bertolini F Spagnolo P. Large cell neuroendocrine carcinoma of the lung: chemotherapy regimen depends on how “large” your diagnostic criteria are. Eur Respir J. (2017) 50(4):1701292. doi: 10.1183/13993003.01292-2017 29074547

[18] Wang H Zhu Y Sun W Yang X Liu XY Chi KW. Clonality analysis for the relationship between the pulmonary combined neuroendocrine carcinoma and “the so-called reported histologic transformation. Cancers (Basel). (2023) 15(23):5649. doi: 10.3390/cancers15235649 38067352 PMC10705092

[19] Yang L Fan Y Lu H. Pulmonary large cell neuroendocrine carcinoma. Pathol Oncol Res. (2022) 28:1610730. doi: 10.3389/pore.2022.1610730 36304941 PMC9592721

[20] Popper H Brcic L. Diagnosis and molecular profiles of large cell neuroendocrine carcinoma with potential targets for therapy. Front Oncol. (2021) 11:655752. doi: 10.3389/fonc.2021.655752 34307132 PMC8293294

[21] Travis WD Rush W Flieder DB Fleming MV Gal AA Koss MN. Survival analysis of 200 pulmonary neuroendocrine tumors with clarification of criteria for atypical carcinoid and its separation from typical carcinoid. Am J Surg Pathol. (1998) 22(8):934–44. doi: 10.1097/00000478-199808000-00003 9706973

[22] Baine MK Rekhtman N. Multiple faces of pulmonary large cell neuroendocrine carcinoma: update with a focus on practical approach to diagnosis. Transl Lung Cancer Res. (2020) 9(3):860–78. doi: 10.21037/tlcr.2020.02.13 PMC7354156 32676352

[23] den Bakker MA Willemsen S Grunberg K Noorduijn LA van Oosterhout MFM van Suylen RJ. Small cell carcinoma of the lung and large cell neuroendocrine carcinoma interobserver variability. Histopathology. (2010) 56(3):356–63. doi: 10.1111/j.1365-2559.2010.03486.x 20459535

[24] Righi L Volante M Rapa I Tavaglione V Inzani F Pelosi G. Mammalian target of rapamycin signaling activation patterns in neuroendocrine tumors of the lung. Endocr Relat Cancer. (2010) 17(4):977–87. doi: 10.1677/erc-10-0157 20817788

[25] Pelosi G Rindi G Travis WD Papotti M. Ki-67 antigen in lung neuroendocrine tumors: unraveling a role in clinical practice. J Thorac Oncol. (2014) 9(3):273–84. doi: 10.1097/jto.0000000000000092 24518085

[26] Rindi G Klersy C Fellegara G Ampollini L Ardizzoni A Campanini N. Grading the neuroendocrine tumors of the lung: an evidence-based proposal. Endocr Relat Cancer. (2014) 21(1):1–16. doi: 10.1530/erc-13-0246 24344249

[27] Pelosi G Massa F Gatti G Righi L Volante M Birocco N. Ki-67 evaluation for clinical decision in metastatic lung carcinoids: A proof of concept. Clin Pathol. (2019) 12:2632010X19829259. doi: 10.1177/2632010x19829259 PMC6477754 31041430

[28] Oka N Kasajima A Konukiewitz B Sakurada A Okada Y Kameya T. Classification and prognostic stratification of bronchopulmonary neuroendocrine neoplasms. Neuroendocrinology. (2020) 110(5):393–403. doi: 10.1159/000502776 31422400

[29] Rekhtman N Pietanza MC Hellmann MD Naidoo J Arora A Won H. Next-generation sequencing of pulmonary large cell neuroendocrine carcinoma reveals small cell carcinoma-like and non-small cell carcinoma-like subsets. Clin Cancer Res. (2016) 22(14):3618–29. doi: 10.1158/1078-0432.ccr-15-2946 PMC4995776 26960398

[30] Derks JL Leblay N Thunnissen E van Suylen RJ den Bakker M Groen HJM. Molecular subtypes of pulmonary large-cell neuroendocrine carcinoma predict chemotherapy treatment outcome. Clin Cancer Res. (2018) 24(1):33–42. doi: 10.1158/1078-0432.ccr-17-1921 29066508

[31] George J Walter V Peifer M Alexandrov LB Seidel D Leenders F. Integrative genomic profiling of large-cell neuroendocrine carcinomas reveals distinct subtypes of high-grade neuroendocrine lung tumors. Nat Commun. (2018) 9(1):1048. doi: 10.1038/s41467-018-03099-x 29535388 PMC5849599

[32] Przygodzki RM Finkelstein SD Langer JC Swalsky PA Fishback N Bakker A. Analysis of p53, K-ras-2, and C-raf-1 in pulmonary neuroendocrine tumors. Correlation with histological subtype and clinical outcome. Am J Pathol. (1996) 148:1531–41. PMC1861560 8623922

[33] Rusch VW Klimstra DS Venkatraman ES. Molecular markers help characterize neuroendocrine lung tumors. Ann Thorac Surg. (1996) 62(3):798–809. doi: 10.1016/s0003-4975(96)00435-3 8784011

[34] Clinical Lung Cancer Genome, P M. Network Genomic. A genomics-based classification of human lung tumors. Sci Transl Med. (2013) 5(209):209ra153. doi: 10.1126/scitranslmed.3006802 PMC4006630 24174329

[35] Milione M Maisonneuve P Grillo F Mangogna A Centonze G Prinzi N. Ki-67 index of 55% Distinguishes two groups of bronchopulmonary pure and composite large cell neuroendocrine carcinomas with distinct prognosis. Neuroendocrinology. (2021) 111(5):475–89. doi: 10.1159/000508376 32365350

[36] Simbolo M Centonze G Giudice L Grillo F Maisonneuve P Gkountakos A. Combined large cell neuroendocrine carcinomas of the lung: integrative molecular analysis identifies subtypes with potential therapeutic implications. Cancers (Basel). (2022) 14(19):4653. doi: 10.3390/cancers14194653 36230576 PMC9562868

[37] Lazaro S Perez-Crespo M Lorz C Bernardini A Oteo M Enguita AB. Differential development of large-cell neuroendocrine or small-cell lung carcinoma upon inactivation of 4 tumor suppressor genes. Proc Natl Acad Sci U.S.A. (2019) 116(44):22300–6. doi: 10.1073/pnas.1821745116 PMC6825275 31611390

[38] Simbolo M Barbi S Fassan M Mafficini A Ali G Vicentini C. Gene expression profiling of lung atypical carcinoids and large cell neuroendocrine carcinomas identifies three transcriptomic subtypes with specific genomic alterations. J Thorac Oncol. (2019) 14(9):1651–61. doi: 10.1016/j.jtho.2019.05.003 31085341

[39] Lou G Yu X Song Z. Molecular profiling and survival of completely resected primary pulmonary neuroendocrine carcinoma. Clin Lung Cancer. (2017) 18(3):e197–201. doi: 10.1016/j.cllc.2016.11.014 28024928

[40] Ando T Kage H Shinozaki-Ushiku A Tatsuno K Tsutsumi S Nagayama K. Composite clonal analysis reveals transition of NSCLC subtypes through accumulation of gene mutations: A case report. JTO Clin Res Rep. (2022) 3(2):100277. doi: 10.1016/j.jtocrr.2022.100277 35199052 PMC8844245

[41] Sakamoto T Arai K Makishima K Yamasaki A. BRAF V600E-mutated combined large cell neuroendocrine carcinoma and adenocarcinoma responding to targeted therapy. BMJ Case Rep. (2021) 14(12):e243295. doi: 10.1136/bcr-2021-243295 PMC8719127 34969785

[42] Zhu Z Liu Y Xu HL Ning HY Xia YM Shen LL. Combined large cell neuroendocrine carcinoma, lung adenocarcinoma, and squamous cell carcinoma: a case report and review of the literature. J Cardiothorac Surg. (2023) 18(1):254. doi: 10.1186/s13019-023-02349-4 37653509 PMC10472660

[43] Igawa S Watanabe R Ito I Murakami H Takahashi T Nakamura Y. Comparison of chemotherapy for unresectable pulmonary high-grade non-small cell neuroendocrine carcinoma and small-cell lung cancer. Lung Cancer. (2010) 68(3):438–45. doi: 10.1016/j.lungcan.2009.07.003 19699548

[44] Hanna N Johnson D Temin S Baker S Jr Brahmer J Ellis PM. Systemic therapy for stage IV non-small-cell lung cancer: american society of clinical oncology clinical practice guideline update. J Clin Oncol. (2017) 35:3484–515. doi: 10.1200/jco.2017.74.6065 28806116

[45] Zacharias J Nicholson AG Ladas GP Goldstraw P. Large cell neuroendocrine carcinoma and large cell carcinomas with neuroendocrine morphology of the lung: prognosis after complete resection and systematic nodal dissection. Ann Thorac Surg. (2003) 75(2):348–52. doi: 10.1016/s0003-4975(02)04118-8 12607637

[46] Roesel C Terjung S Weinreich G Gauler T Theegarten D Stamatis G. A single-institution analysis of the surgical management of pulmonary large cell neuroendocrine carcinomas. Ann Thorac Surg. (2016) 101(5):1909–14. doi: 10.1016/j.athoracsur.2015.12.009 26897321

[47] Tang H Wang HY Xi SY He CY Chang YX Wang QM. Perioperative chemotherapy with pemetrexed and cisplatin for pulmonary large-cell neuroendocrine carcinoma: a case report and literature review. Onco Targets Ther. (2018) 11:2557–63. doi: 10.2147/OTT.S160565 PMC5944445 29765234

[48] Sarkaria IS Iyoda A Roh MS Sica G Kuk D Sima CS. Neoadjuvant and adjuvant chemotherapy in resected pulmonary large cell neuroendocrine carcinomas: a single institution experience. Ann Thorac Surg. (2011) 92(4):1180–6. doi: 10.1016/j.athoracsur.2011.05.027 21867986

[49] Welter S Aigner C Roesel C. The role of surgery in high grade neuroendocrine tumours of the lung. J Thorac Dis. (2017) 9(Suppl 15):S1474–83. doi: 10.21037/jtd.2017.01.60 PMC5690951 29201450

[50] Kumar P Herndon J 2nd Langer M Kohman LJ Elias AD Kass FC. Patterns of disease failure after trimodality therapy of nonsmall cell lung carcinoma pathologic stage IIIA (N2). Analysis of Cancer and Leukemia Group B Protocol 8935. Cancer. (1996) 77(11):2393–9. doi: 10.1002/(sici)1097-0142(19960601)77:11<2393::aid-cncr31>3.0.co;2-q 8635112

[51] Kujtan L Muthukumar V Kennedy KF Davis JR Masood A Subramanian J. The role of systemic therapy in the management of stage I large cell neuroendocrine carcinoma of the lung. J Thorac Oncol. (2018) 13(5):707–14. doi: 10.1016/j.jtho.2018.01.019 29391287

[52] Yilmaz A Duyar SS Cakir E Aydin E Demirag F Karakaya J. Clinical impact of visceral pleural, lymphovascular and perineural invasion in completely resected non-small cell lung cancer. Eur J Cardiothorac Surg. (2011) 40(3):664–70. doi: 10.1016/j.ejcts.2010.12.059 21334917

[53] Saji H Tsuboi M Matsubayashi J Miyajima K Shimada Y Imai K. Clinical response of large cell neuroendocrine carcinoma of the lung to perioperative adjuvant chemotherapy. Anticancer Drugs. (2010) 21(1):89–93. doi: 10.1097/cad.0b013e328330fd79 19770636

[54] Iyoda A Hiroshima K Moriya Y Takiguchi Y Sekine Y Shibuya K. Prospective study of adjuvant chemotherapy for pulmonary large cell neuroendocrine carcinoma. Ann Thorac Surg. (2006) 82(5):1802–7. doi: 10.1016/j.athoracsur.2006.05.109 17062251

[55] Kim KW Kim HK Kim J Shim YM Ahn MJ Choi YL. Outcomes of curative-intent surgery and adjuvant treatment for pulmonary large cell neuroendocrine carcinoma. World J Surg. (2017) 41(7):1820–7. doi: 10.1007/s00268-017-3908-8 28204910

[56] Iyoda A Hiroshima K Moriya Y Iwadate Y Takiguchi Y Uno T. Postoperative recurrence and the role of adjuvant chemotherapy in patients with pulmonary large-cell neuroendocrine carcinoma. J Thorac Cardiovasc Surg. (2009) 138(2):446–53. doi: 10.1016/j.jtcvs.2008.12.037 19619794

[57] Sun JM Ahn MJ Ahn JS Um SW Kim H Kim HK. Chemotherapy for pulmonary large cell neuroendocrine carcinoma: similar to that for small cell lung cancer or non-small cell lung cancer? Lung Cancer. (2012) 77(2):365–70. doi: 10.1016/j.lungcan.2012.04.009 22579297

[58] Niho S Kenmotsu H Sekine I Ishii G Ishikawa Y Noguchi M. Combination chemotherapy with irinotecan and cisplatin for large-cell neuroendocrine carcinoma of the lung: a multicenter phase II study. J Thorac Oncol. (2013) 8(7):980–4. doi: 10.1097/jto.0b013e31828f6989 23774385

[59] Kenmotsu H Niho S Ito T Ishikawa Y Noguchi M Tada H. A pilot study of adjuvant chemotherapy with irinotecan and cisplatin for completely resected high-grade pulmonary neuroendocrine carcinoma (large cell neuroendocrine carcinoma and small cell lung cancer). Lung Cancer. (2014) 84(3):254–8. doi: 10.1016/j.lungcan.2014.03.007 24679951

[60] Fujiwara Y Sekine I Tsuta K Ohe Y Kunitoh H Yamamoto N. Effect of platinum combined with irinotecan or paclitaxel against large cell neuroendocrine carcinoma of the lung. Jpn J Clin Oncol. (2007) 37(7):482–6. doi: 10.1093/jjco/hym053 17652109

[61] Tokito T Kenmotsu H Watanabe R Ito I Shukuya T Ono A. Comparison of chemotherapeutic efficacy between LCNEC diagnosed using large specimens and possible LCNEC diagnosed using small biopsy specimens. Int J Clin Oncol. (2014) 19(1):63–7. doi: 10.1007/s10147-012-0509-2 23250620

[62] Zhuo M Guan Y Yang X Hong LZ Wang YQ Li ZW. The prognostic and therapeutic role of genomic subtyping by sequencing tumor or cell-free DNA in pulmonary large-cell neuroendocrine carcinoma. Clin Cancer Res. (2020) 26(4):892–901. doi: 10.1158/1078-0432.ccr-19-0556 31694833 PMC7024651

[63] Wang Z Wu Y Lu T Xu Y Chen MJ Zhong W. The outcomes of different regimens depend on the molecular subtypes of pulmonary large-cell neuroendocrine carcinoma: A retrospective study in China. Cancer Med. (2024) 13(1):e6834. doi: 10.1002/cam4.6834 38180312 PMC10807557

[64] Le Treut J C Sault M Lena H Souquet PJ Vergnenegre A Caer HL. Multicentre phase II study of cisplatin-etoposide chemotherapy for advanced large-cell neuroendocrine lung carcinoma: the GFPC 0302 study. Ann Oncol. (2013) 24(6):1548–52. doi: 10.1093/annonc/mdt009 23406729

[65] Filosso PL Fontana EC Ruffini E. Large-cell neuroendocrine carcinoma and combined large-cell neuroendocrine carcinoma: 2 characters in search of an author. Eur J Cardiothorac Surg. (2022) 62(2):ezac176. doi: 10.1093/ejcts/ezac176 35277717

[66] Yoshimura M Seki K Bychkov A Fukuoka J. Molecular pathology of pulmonary large cell neuroendocrine carcinoma: novel concepts and treatments. Front Oncol. (2021) 11:671799. doi: 10.3389/fonc.2021.671799 33968782 PMC8100606

[67] Corbett V Arnold S Anthony L Chauhan A. Management of large cell neuroendocrine carcinoma. Front Oncol. (2021) 11:653162. doi: 10.3389/fonc.2021.653162 34513663 PMC8432609

[68] Rossi A Maio MD Chiodini P Rudd RM Okamoto H Skarlos DV. Carboplatin- or cisplatin-based chemotherapy in first-line treatment of small-cell lung cancer: the COCIS meta-analysis of individual patient data. J Clin Oncol. (2012) 30(14):1692–8. doi: 10.1200/jco.2011.40.4905 22473169

[69] Derks JL Suylen RJ Thunnissen E den Bakker MA Groen HJ Smit EF. Chemotherapy for pulmonary large cell neuroendocrine carcinomas: does the regimen matter? Eur Respir J. (2017) 49(6):1601838. doi: 10.1183/13993003.01838-2016 28572122 PMC5898951

[70] Naidoo J Santos-Zabala ML Iyriboz T Woo KM Sima CS Fiore JJ. Large cell neuroendocrine carcinoma of the lung: clinico-pathologic features, treatment, and outcomes. Clin Lung Cancer. (2016) 17(5):e121–9. doi: 10.1016/j.cllc.2016.01.003 PMC5474315 26898325

[71] Wang Y Shen YH Ma S Zhou JY. A marked response to icotinib in a patient with large cell neuroendocrine carcinoma harboring an EGFR mutation: A case report. Oncol Lett. (2015) 10(3):1575–8. doi: 10.3892/ol.2015.3405 PMC4533524 26622712

[72] Aroldi F Bertocchi P Meriggi F Abeni C Ogliosi C Rota L. Tyrosine kinase inhibitors in EGFR-mutated large-cell neuroendocrine carcinoma of the lung? A Case Rep Case Rep Oncol. (2014) 7(2):478–83. doi: 10.1159/000365413 PMC4154195 25202262

[73] De Pas TM Giovannini M Manzotti M Trifiro G Toffalorio F Catania C. Large-cell neuroendocrine carcinoma of the lung harboring EGFR mutation and responding to gefitinib. J Clin Oncol. (2011) 29(34):e819–22. doi: 10.1200/JCO.2011.36.2251 22042963

[74] Yoshida Y Ota S Murakawa T Takai D Nakajima J. Combined large cell neuroendocrine carcinoma and adenocarcinoma with epidermal growth factor receptor mutation in a female patient who never smoked. Ann Thorac Cardiovasc Surg. (2014) 20 Suppl:582–4. doi: 10.5761/atcs.cr.12.02217 23518631

[75] Yanagisawa S Morikawa N Kimura Y Nagano Y Murakami K Tabata T. Large-cell neuroendocrine carcinoma with epidermal growth factor receptor mutation: possible transformation of lung adenocarcinoma. Respirology. (2012) 17(8):1275–7. doi: 10.1111/j.1440-1843.2012.02258.x 22943430

[76] Wang S Wu X Zhao JZ Chen HY Zhang Z Wang MY. Next-generation sequencing identified a novel crizotinib-sensitive PLB1-ALK rearrangement in lung large-cell neuroendocrine carcinoma. Clin Lung Cancer. (2021) 22(3):e366–70. doi: 10.1016/j.cllc.2020.05.026 32651063

[77] Lim CA Banyi N Tucker T Ionescu DN Melosky B. A case of ALK-rearranged combined lung adenocarcinoma and neuroendocrine carcinoma with diffuse bone metastasis and partial response to alectinib. Curr Oncol. (2022) 29(2):848–52. doi: 10.3390/curroncol29020072 PMC8870951 35200571

[78] Omachi N Shimizu S Kawaguchi T Tezuka K Kanazu M Tamiya A. A case of large-cell neuroendocrine carcinoma harboring an EML4-ALK rearrangement with resistance to the ALK inhibitor crizotinib. J Thorac Oncol. (2014) 9(6):e40–2. doi: 10.1097/jto.0000000000000103 24828670

[79] Hayashi N Fujita A Saikai T Takabatake H Sotoshiro M Sekine K. Large cell neuroendocrine carcinoma harboring an anaplastic lymphoma kinase (ALK) rearrangement with response to alectinib. Intern Med. (2018) 57(5):713–6. doi: 10.2169/internalmedicine.9368-17 PMC5874345 29151522

[80] Hoton D Humblet Y Libbrecht L. Phenotypic variation of an ALK-positive large-cell neuroendocrine lung carcinoma with carcinoid morphology during treatment with ALK inhibitors. Histopathology. (2018) 72(4):707–10. doi: 10.1111/his.13388 28888040

[81] van Moorsel CJ Pinedo HM Veerman G Guechev A Smid K Loves WJ. Combination chemotherapy studies with gemcitabine and etoposide in non-small cell lung and ovarian cancer cell lines. Biochem Pharmacol. (1999) 57(4):407–15. doi: 10.1016/s0006-2952(98)00316-5 9933029

[82] Gandara DR Chansky K Albain KS Leigh BR Gaspar LE Lara PN Jr. Consolidation docetaxel after concurrent chemoradiotherapy in stage IIIB non-small-cell lung cancer: phase II Southwest Oncology Group Study S9504. J Clin Oncol. (2003) 21(10):2004–10. doi: 10.1200/jco.2003.04.197 12743155

[83] Pusuluri A Wu D Mitragotri S. Immunological consequences of chemotherapy: Single drugs, combination therapies and nanoparticle-based treatments. J Control Release. (2019) 305:130–54. doi: 10.1016/j.jconrel.2019.04.020 31004668

[84] Pomeroy AE Schmidt EV Sorger PK Palmer AC. Drug independence and the curability of cancer by combination chemotherapy. Trends Cancer. (2022) 8(11):915–29. doi: 10.1016/j.trecan.2022.06.009 PMC9588605 35842290

[85] Frei E 3rd Freireich EJ. Progress and perspectives in the chemotherapy of acute leukemia. Adv Chemother. (1965) 2:269–98. doi: 10.1016/b978-1-4831-9930-6.50011-3 5319962

[86] Pritchard JR Lauffenburger DA Hemann MT. Understanding resistance to combination chemotherapy. Drug Resist Update. (2012) 15(5-6):249–57. doi: 10.1016/j.drup.2012.10.003 PMC3975170 23164555

[87] Wang HY Yan LH Zhu YL Sun W Yang X Liu XY. Exploring the molecular features and genetic prognostic factors of pulmonary high-grade neuroendocrine carcinomas. Hum Pathol. (2023) 142:81–9. doi: 10.1016/j.humpath.2023.09.002 37742943

[88] May MS Kinslow CJ Adams C Saqi A Shu CA Chaudhary KR. Outcomes for localized treatment of large cell neuroendocrine carcinoma of the lung in the United States. Transl Lung Cancer Res. (2021) 10(1):71–9. doi: 10.21037/tlcr-20-374 PMC7867769 33569294

[89] Jiang YZ Lei C Zhang XF Cui YG Che KY Shen HC. Double-edged role of radiotherapy in patients with pulmonary large-cell neuroendocrine carcinoma. J Cancer. (2019) 10(25):6422–30. doi: 10.7150/jca.32446 PMC6856741 31772675

[90] Prelaj A Rebuzzi SE Bene GD Berrios JRG Emiliani A De Filioois L. Evaluation of the efficacy of cisplatin-etoposide and the role of thoracic radiotherapy and prophylactic cranial irradiation in LCNEC. ERJ Open Res. (2017) 3(1):00128–2016. doi: 10.1183/23120541.00128-2016 PMC5370316 28382303

[91] Rieber J Schmitt J Warth A Muley T Kappes Js Eichhorn F. Outcome and prognostic factors of multimodal therapy for pulmonary large-cell neuroendocrine carcinomas. Eur J Med Res. (2015) 20(1):64. doi: 10.1186/s40001-015-0158-9 26272455 PMC4536693

[92] Sherman S Rotem O Shochat T Zer A Moore A Dudnik E. Efficacy of immune check-point inhibitors (ICPi) in large cell neuroendocrine tumors of lung (LCNEC). Lung Cancer. (2020) 143:40–6. doi: 10.1016/j.lungcan.2020.03.008 32203769

[93] Dudnik E Kareff S Moskovitz M Kim C Liu SV Lobachov A. Real-world survival outcomes with immune checkpoint inhibitors in large-cell neuroendocrine tumors of lung. J Immunother Cancer. (2021) 9(2):e001999. doi: 10.1136/jitc-2020-001999 33597218 PMC7893659

[94] Oda R Okuda K Yamashita Y Sakane T Tatematsu T Yokota K. Long-term survivor of pulmonary combined large cell neuroendocrine carcinoma treated with nivolumab. Thorac Cancer. (2020) 11(7):2036–9. doi: 10.1111/1759-7714.13471 PMC7327674 32379390

[95] Xu J Feng Q Chen Y Liu XL Jiang O. Complete remission of combined pulmonary large cell neuroendocrine carcinoma: a case report. J Int Med Res. (2021) 49(11):3000605211055387. doi: 10.1177/03000605211055387 34738481 PMC8573517

[96] Tsutsumi R Kataoka N Kunimatsu Y Sato I Tanimura M Nakano T. Atezolizumab in combination with carboplatin plus nab-paclitaxel for managing combined large-cell neuroendocrine carcinoma: A case report. Respirol Case Rep. (2022) 10(7):e0989. doi: 10.1002/rcr2.989 35685848 PMC9171681

